# Identification of a novel anoikis-related gene signature to predict prognosis and tumor microenvironment in intrahepatic cholangiocarcinoma carcinoma

**DOI:** 10.1007/s12672-025-02840-5

**Published:** 2025-06-10

**Authors:** Xuan Zhou, Bai Wei, Yan Wang, Mingjie Liu, Xiangru Guo, Yuting Duan

**Affiliations:** Department of Oncology, Liyuan Hospital, Tongji Medical College, Huazhong University of Science and Technology, 39 Yanhu Avenue, Wuchang, Wuhan, 430077 Hubei People’s Republic of China

## Abstract

**Background:**

Intrahepatic cholangiocarcinoma is a malignant tumor of hepatobiliary epithelial cells. In recent years, its incidence has gradually increased. It has a very high fatality rate and low survival rate, and the existing predictive factors for intrahepatic cholangiocarcinoma are unclear. The role of anoikis, a form of programmed cell death, in intrahepatic cholangiocarcinoma is not fully understood. This study focuses on identifying and analyzing anoikis-related differentially expressed genes in intrahepatic cholangiocarcinoma, aiming to enhance our understanding of potential treatment strategies and prognosis of intrahepatic cholangiocarcinoma.

**Methods:**

In our study, we employed a clustering algorithm to classify samples from The Cancer Genome Atlas (TCGA) based on differentially expressed overlapping anoikis-related genes. Subsequently, we utilized Weighted Gene Co-expression Network Analysis (WGCNA) to identify highly correlated genes and constructed a prognostic risk model based on univariate Cox proportional hazard regression. We validated the model's reliability using external datasets from the International Cancer Genome Consortium (ICGC) and the Gene Expression Omnibus (GEO). Finally, we used the CIBERSORT algorithm to investigate the correlation between risk scores and immune infiltration.

**Results:**

The results showed that the TCGA cohort could be divided into 2 subgroups, among which subgroup B had a lower survival probability. We identified three prognostic genes (EGF, BNIP3, TDGF1) associated with anorexia. The prognostic risk model effectively predicted overall survival and was validated in ICGC and GEO data sets. Furthermore, there were significant correlations between infiltrating immune cells and prognostic genes and risk scores.

**Conclusion:**

We identified subgroups and prognostic genes associated with ICCA dysregulation, which are important for understanding the treatment and prognosis of ICCA.

## Introduction

Intrahepatic cholangiocarcinoma (ICCA) is the second most common primary liver cancer and one of the deadliest malignant tumors in the majority of patients. Only about 20–30% of patients are eligible for surgical resection, which is considered a potential treatment approach [1]. The early clinical symptoms of ICCA patients are often not obvious, resulting in missed opportunities for surgical intervention after discovery [2]. In recent years, many studies have provided new insights into the early diagnosis and prognosis of ICCA, TGF-β, PDGF-D, ABT-199, and others have recently been found to be key factors influencing the recruitment and expansion of ICCA tumor cells [3–6], proteins within serum extracellular vesicles have also been found to be excellent biomarkers for diagnosing ICCA [7]. Additionally, the neutrophil-to-eosinophil ratio, as a promising and non-invasive biomarker, has shown good predictive value in cancer patients [8]. However, the current mechanism is still limited to effectively predict the prognosis of ICCA.

Anoikis is a feature of programmed cell death, triggered when cells detach from their surrounding extracellular matrix. It plays a crucial role in normal development and maintaining tissue homeostasis, while resistance to anoikis is pivotal in the invasion and metastasis of cancer [9]. Highly metastatic colorectal cancer cells exhibit a greater degree of resistance to anoikis compared to normal intestinal epithelial cells, with the α2β1/α5β1 integrin/EGFR pathway supporting cell survival and stimulating resistance to anoikis in colorectal cancer [10]. Besides, the secretion of platelet-derived growth factor-BB (PDGFB) from gastric cancer cells resistant to anoikis establishes a C/EBPβ-PDGFB-PDGFRβ-MAPK feedback loop with vascular endothelial cells (ECs), promoting angiogenesis and metastasis in gastric cancer [11]. In breast cancer collagen XIII/β1 integrin [12], in PDAC COL11A1/Akt/Cyclic AMP response-element binding protein (CREB) [13], and in hepatoma collagen IV/integrin supports resistance to anoikis through B-cell lymphoma (BCL) family proteins and associated downstream pathways [14]. Despite studies confirming the association between anoikis and cancer progression and metastasis, there has been limited exploration of prognostic models for intrahepatic cholangiocarcinoma based on anoikis-related genes.

The therapeutic landscape in cancer has evolved significantly, particularly with the emergence of targeted therapies and immunotherapy approaches [15, 16]. In recent years, immune checkpoint inhibitors such as PD-1/PD-L1 have made significant progress in tumor treatment, especially in the treatment of intrahepatic cholangiocarcinoma (ICCA), where approximately 30% of patients have PD-L1 expression [17]. The combination of durvalumab (anti-PD-L1) with gemcitabine plus cisplatin has demonstrated significant survival benefits in advanced biliary tract cancer [18]. However, the immunotherapy response rate of ICCA is relatively low, and the efficacy of monotherapy needs to be improved. Given that approximately 60–70% of surgically resected biliary tract cancer patients experience disease relapse, studying the interaction mechanism between tumors and immunity in ICCA has become a key entry point to solve existing immunotherapy problems [19]. In ICCA, strategies to reverse the anoikis-resistant phenotype of tumor cells to inhibit cancer metastasis may have potential value in treating patients with local metastasis and potential vascular invasion. Some studies have clarified the relationship between tumor resistance to anoikis and immunity, revealing how anoikis affects the tumor immune microenvironment and the anoikis resistance of cancer cells. For example, studies have found that inactivating IL1RAP can promote anoikis and inhibit Ewing sarcoma cell metastasis, indicating that anoikis-targeted therapy has great potential in cancer treatment [20]. In addition, exploring the role of anoikis in ICCA will not only help more accurate prognostic stratification of patients but also provide insights for the development of new therapeutic targets. This may enhance the effectiveness of combination therapy with cancer vaccines and immunotherapy in ICCA. For example, risk models based on anoikis-related long noncoding RNAs (lncRNAs) have been developed to predict bladder cancer prognosis and immunotherapy response. In hepatocellular carcinoma (HCC), anoikis-based molecular subtypes have also been proposed, which not only contribute to personalized prognostic stratification of HCC patients but also provide a basis for identifying the best candidate tumor vaccines and enhancing immunotherapy strategies [21]. A blueprint is provided. In bladder cancer, Ascore is proposed as a new, robust prognostic biomarker that can help enhance immunotherapy decision-making and provide new tools for customized treatment approaches [22]. Therefore, exploring the role of anoikis in ICCA will provide valuable insights into patient prognosis stratification and the development of new therapeutic targets, which may also potentially enhance combination therapy with cancer vaccines and immunotherapy in ICCA.

This study aims to explore the mechanisms associated with anoikis resistance in intrahepatic cholangiocarcinoma (ICCA). We employed an unsupervised clustering algorithm to classify samples from patients with ICCA based on differentially expressed overlapping anoikis-related genes in The Cancer Genome Atlas (TCGA) dataset. Subsequently, we utilized Weighted Gene Co-expression Network Analysis (WGCNA) to identify highly correlated genes associated with anoikis resistance and constructed a prognostic risk model based on univariate Cox proportional hazard regression. To validate the reliability of the model, external datasets from the International Cancer Genome Consortium (ICGC) and the Gene Expression Omnibus (GEO) were used for validation. Additionally, we employed the CIBERSORT algorithm to investigate the relationship between risk scores and immune infiltration. This model has the potential to predict the prognosis of ICCA patients and provides insights into the immune tumor microenvironment (TME) of ICCA. This risk model can further be used to promote precision therapy for ICCA, such as immunotherapy.

## Materials and methods

### Identification of anoikis-related genes in ICC

We obtained RNA sequencing data and clinical information of 41 ICC patients from the TCGA database, including 32 LUAD samples and 9 noncancerous adjacent samples (NAT). A total of 653 ARGs were retrieved from the GeneCards database and Harmonizome database, and genes with correlation scores > 0.4 were selected for inclusion in our study. In addition, we obtained the ICC gene expression dataset GSE53870 from the GEO database, which includes tumor tissues from ICC samples of 63 patients. We installed the limma and pheatmap packages in R and read the gene expression data and gene list, filtered low-expressed genes, used the Wilcoxon rank sum test to calculate the difference table between the normal group and the tumor group, and finally output the differentially expressed gene table and pairs Visualize the results.

### Cox regression analysis

Survival analysis involves reading and processing gene expression and survival data, so we performed Cox regression analysis on each gene and outputted single-factor results such as HR, HR.95L, HR.95H, and p-value.

### weighted gene coexpression network analysis (WGCNA)

GCNA was utilized to identify highly correlated gene modules in TCGA-ICCA. To read the gene expression and Cox survival analysis result files, we found the intersection of genes in the two input files, calculated the pairwise correlations between genes, and prepared an edge list for the network graph.

### Consensus clustering analysis of anoikis-related genes

Based on the overlapping anoikis-related genes and highly correlated module genes in WGCNA, we performed cluster analysis on the TCGA-ICCA dataset, setting the maximum number of clusters to 9. We utilized the ConsensusClusterPlus function for the analysis, setting various parameters, including the number of repetitions (reps), the size of the sample subset (pItem), the size of the feature subset (pFeature), the clustering algorithm (clusterAlg), the distance metric (distance), etc. The configuration of these parameters was to ensure the accuracy and stability of the clustering results, and finally output the clustering results. Subsequently, we read in the gene expression data and the sample classification result files, performed calculations for PCA, UMAP, and tSNE dimensionality reduction methods, and plotted visualization graphs.

### Functional enrichment analysis

We imported the expression data files and immune gene set files through R, conducted the revised ssGSEA analysis, and read the clustering result files. We merged the clustering results with the immune cell scores, plotted boxplots, performed statistical analysis, and exported the graphical outputs.

### Construction of anoikis-related prognostic risk model

We merged gene expression profiles with survival data for further analysis. We utilized LASSO regression for gene selection and trained a Cox proportional hazards model on the training dataset to predict survival rates, which were then evaluated using the test dataset. The model was considered to have good predictive performance if the p-value was less than 0.01, the area under the ROC curve was greater than 0.68, and the p-values for survival differences between high- and low-risk groups in both training and testing were less than 0.05 and 0.01, respectively. Additionally, we employed the ggplot2, ggalluvial, dplyr, and ggpubr packages in R to create boxplots and alluvial diagrams, combining them into an integrated visualization. Through this visualization, we could better understand the relationship between clustering results and risk scores and further explore the impact of these variables on patient survival status.

### Survival analysis

To perform survival analysis, we installed and loaded the Survival and survminer packages. We then defined a function named "bioSurvival" that read the input files, divided them into high-risk and low-risk categories, and calculated the survival differences between these groups. We utilized the survdiff function to calculate the p-value of the differences; if the p-value was less than 0.001, it was displayed as "*p* < 0.001," or it was shown as "*p* = " followed by the calculated result (rounded to three decimal places).

Next, we used the survfit function to compute the survival curve data and the ggsurvplot function to generate the survival curve plots. We proceeded to call the bioSurvival function three times, providing different input and output file paths to generate survival curve plots for the training set, test set, and the entire dataset. Finally, we employed the pheatmap package to create heatmaps based on risk scores, illustrating the correlation between high-risk status and low-risk status with gene expression.

### Construction and evaluation of the nomogram containing anoi kis-related genes signature

In the entire ICC cohort, univariate and multivariate Cox regression analyses were conducted to identify independent prognostic predictors from the risk score calculated from the signature and several clinicopathological factors, including age, gender, TNM stage, T stage, N stage, and M stage. A nomogram based on identified independent variable factors was established using the “rms” R package. Then, the discrimination and calibration of the nomogram were assessed in the entire CHOL dataset by the ROC and calibration curves. Subsequently, we used the decision curve analysis (DCA) to evaluate the clinical usefulness of the nomogram.

### Tumor immune analysis

To explore the potential biological mechanism and functions of the selected ARGs in signature, we performed gene ontology (GO) and Kyoto Encyclopedia of Genes and Genomes (KEGG) enrichment analyses for the ARGs, the functions or pathways with *p* value < 0.05 were defined as significantly enriched. Functional enrichment analysis was performed using the “clusterProfiler” R package. Furthermore, for exploring the relationship between risk scores calculated from these ARGs and the immune cell abundance, immune function, and immune checkpoints, we used the immune landscape of ICC to compare two risk cohorts using the “limma” R package.

### Tumor immune single cell hub database

An extensive single-cell RNA-seq database devoted to the TME is available online under the name Tumor Immune Single-Cell Hub(TISCH; http://tisch.comp-genomics.org, accessed on 20 August 2023). Utilizing this database, comprehensive research on TME heterogeneity in diverse data sets and cell types was done.

### Statistical analysis

The Kruskal–Wallis test was used to compare data from more than two groups, and Wilcoxon was used to compare data from two groups. Kaplan–Meier log-rank test was used to evaluate each survival curve. A chi-square test was performed to correlate risk score subgroups with somatic mutation frequency, and Spearman analysis was performed to calculate the correlation coefficient. The results of the CIBERSORT algorithm with p < 0.05 were used for further analysis. p < 0.05 was considered statistically significant. R software was used for all statistical analyses.

## Results

### Identification of differentially expressed overlapping anoikis-related genes

**Fig. 1:**
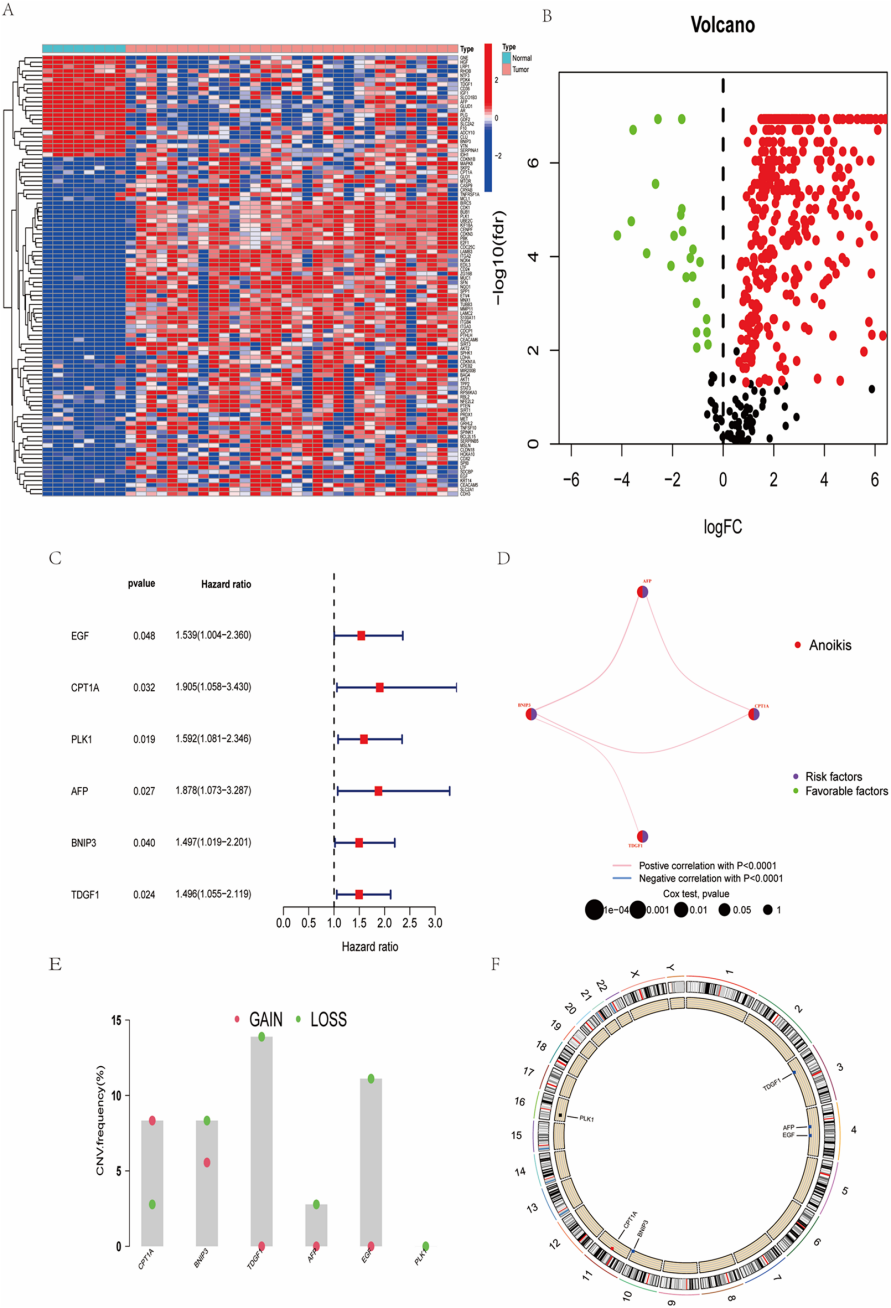
**A** A heatmap of differentially expressed genes, illustrating the expression patterns of these genes across samples. **B** A volcano plot visualizing the significance of differentially expressed genes, where red indicates significantly upregulated genes and green indicates significantly downregulated genes. The x-axis represents logFC (logarithmic fold change in expression), while the y-axis represents -logFDR (negative logarithm of the false discovery rate). **C** The forest plot's right side presents the Hazard Ratio and the 95% confidence interval for each gene, reflecting their estimated values and confidence. The vertical dashed line (black) at Hazard Ratio 1 serves as the reference line, indicating genes with minimal impact on survival rates. Each point on the plot represents a gene's Hazard Ratio, with red denoting values above 1, signifying higher survival risk, and blue indicating values below 1, associated with lower survival risk. **D** Network diagram: illustrates the correlation among genes and their association with survival analysis results. Each node represents a gene, where the size and color denote the significance and attributes of their survival analysis results. The thickness and color of the edges reflect the correlation between genes. **E** The graph displays the CNV frequency, representing both gains and losses. The X-axis denotes different genomic regions, while the Y-axis represents the percentage of CNV frequency. Each bar indicates the maximum frequency of CNV in each region. Points overlaid on the bars depict the frequencies of gains and losses. **F** The scatter plot represents the distribution of Copy Number Variation (CNV) data. Each point on the scatter plot represents the position of CNV on the chromosome

To identify differentially expressed overlapping anoikis-related genes, we first identified the differentially expressed genes (DEGs) in intrahepatic cholangiocarcinoma (ICC) from TCGA. The results revealed 417 genes with differential expression (Fig. 1A, B). Subsequently, we discovered 653 anoikis-related genes from the GeneCards database and ultimately selected 129 differentially expressed overlapping anoikis-related genes for further analysis.

### Construction of the ARGs signature

In order to establish more precise characteristics of anoikis-related genes (ARGs), we conducted univariate regression analysis to identify 5 prognostic-related genes from the pool of 129 DEGs (Fig. 1C). Subsequently, a risk network plot was constructed for these five genes, revealing positive correlations in their risk coefficients (Fig. 1D). Mutation analysis of the 5 prognostic-related genes revealed copy number variations, with PLK1 showing complete copy number loss (Fig. 1E). A copy number circos plot (Fig. 1F) further elucidated the genomic location and copy number variations of the genes across chromosomes.

**Fig. 2:**
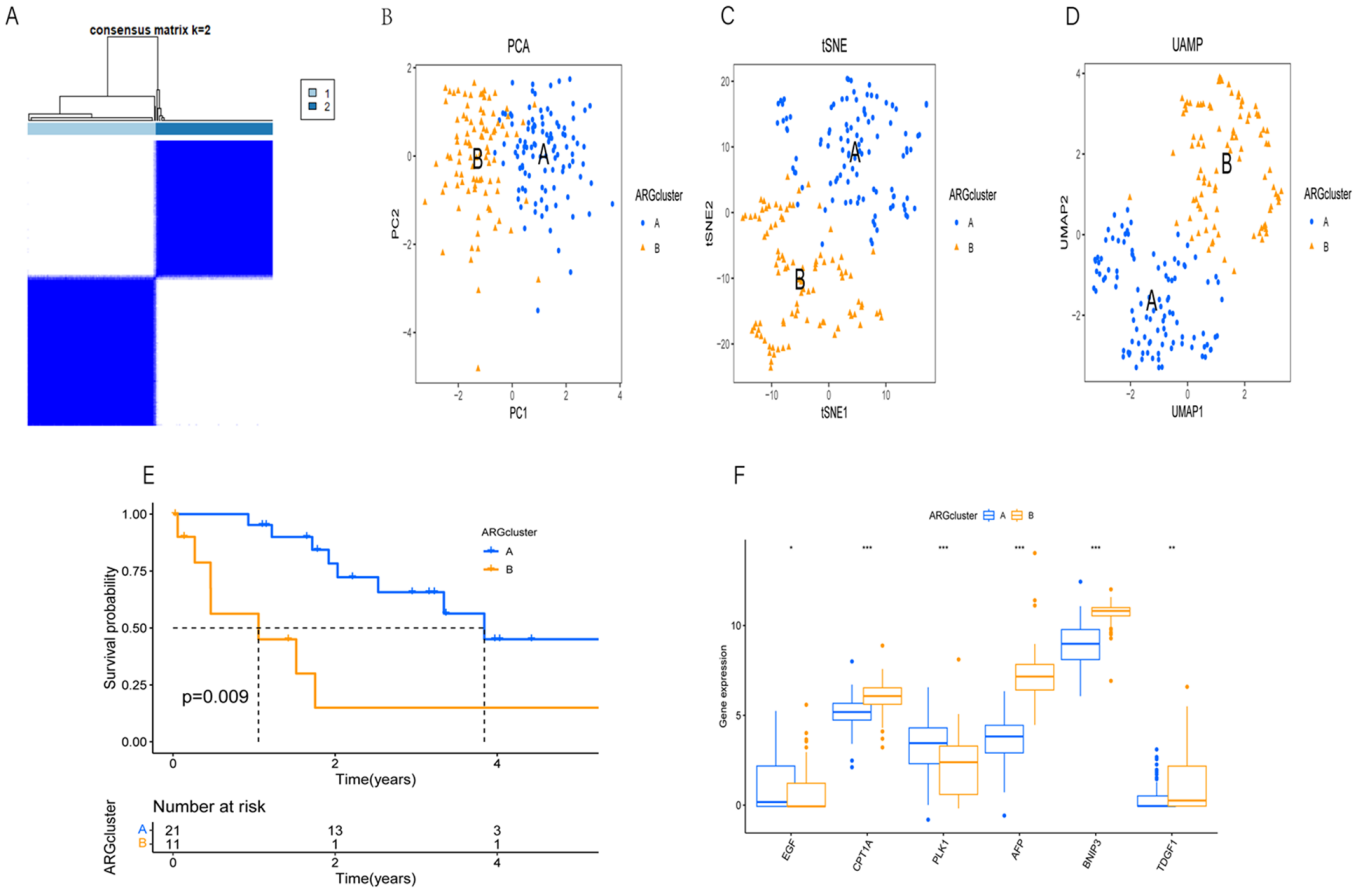
**A** The consensus matrix heatmap defines two gene clusters. The expression k = 2 indicates that two clusters are classified. **B**–**D** The PCA plot illustrates the distribution of samples in the PCA space, where each point represents a sample, and its color and shape indicate the sample's cluster membership, The UMAP plot displays the distribution of samples in the UMAP space, with colors and shapes similarly indicating sample clusters., The tSNE plot also depicts the sample distribution in the tSNE space, utilizing colors and shapes to represent clustering information. **E** In the survival curve plot, the horizontal axis represents time (years), and the vertical axis represents the survival probability. Different colors represent different subtypes, and the curves represent the survival curves of each subtype, with the shaded area indicating the confidence interval. The risk table below displays the number of events and the risk percentage at each time point. **F** The boxplot displays the expression level differences of different genes across various clusters (ARGcluster) and annotates the significance levels

To identify anoikis-related subgroups, unsupervised clustering was performed using the consensus clustering algorithm based on the expression of overlapping anoikis-related genes in the TCGA-LIHC dataset. Optimal clustering was observed at k = 2, delineating subgroups A and B (Fig. 2A). Principal component analysis (PCA) demonstrated a distinct separation between subgroups A and B (Fig. 2B). Additionally, t-distributed stochastic neighbor embedding (t-SNE) and uniform manifold approximation and projection (UMAP) analyses validated these findings (Fig. 2C, D). Survival curve analysis indicated higher survival rates in subgroup A compared to subgroup B (Fig. 2E). Furthermore, a boxplot was used to illustrate the expression levels of anoikis-related genes between the two subgroups (Fig. 2F). These findings underscore the potential of TCGA-LIHC for classification based on anoikis-related genes.

### Visual analysis of clinical variables contrasting groups A and B

**Fig. 3:**
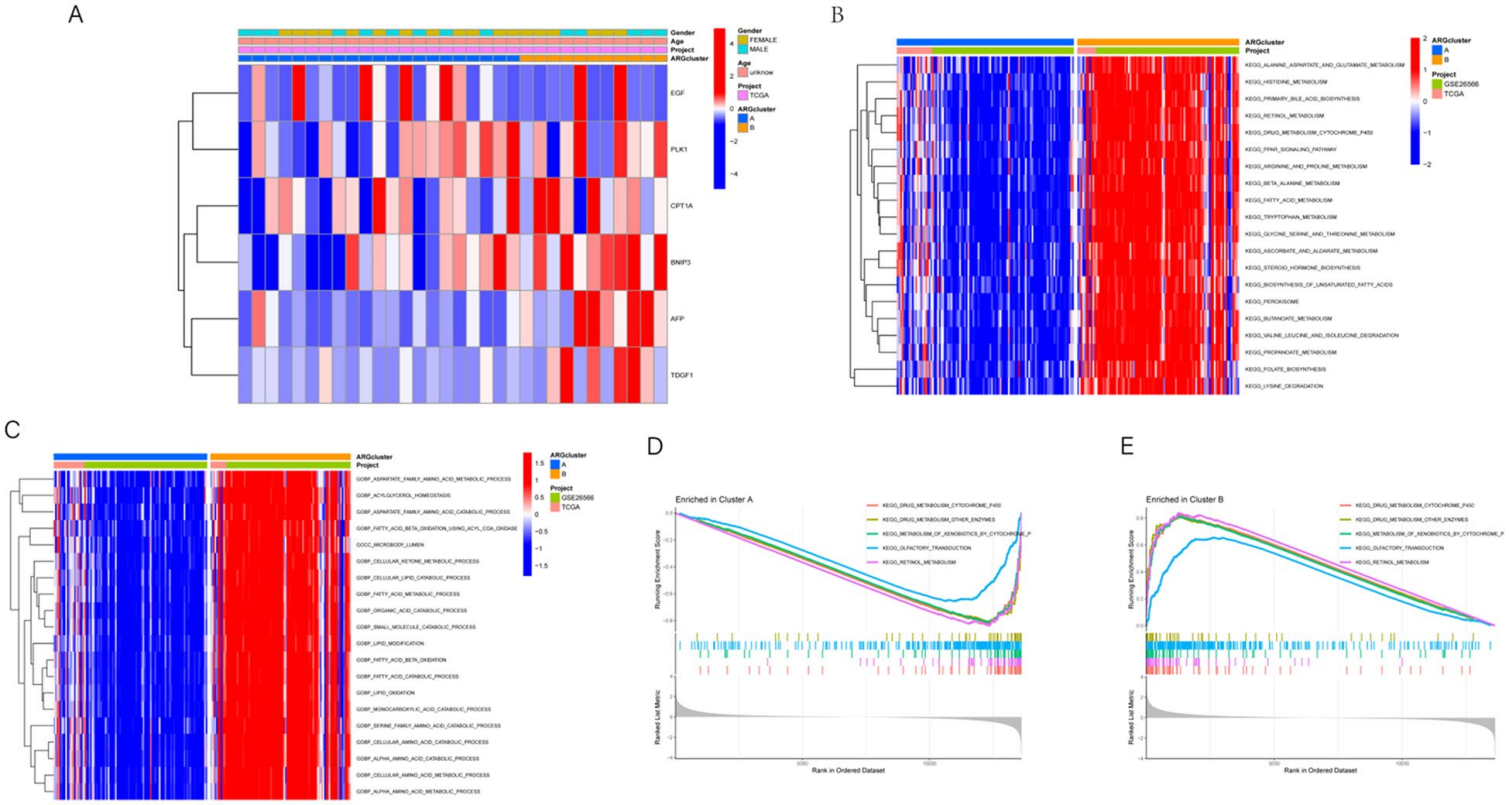
**A** The heatmap illustrates patterns between gene expression and clustering. Rows represent genes, while columns represent samples. The intensity of colors reflects the expression levels of genes across different samples. Both rows and columns are clustered, grouping genes and samples with similar expression patterns together. **B**, **C** The heatmap of differential pathways illustrates the significant differences between the two phenotypic groups. Rows represent genes from the gene set, while columns represent samples. The color intensity reflects the expression levels of genes across different samples. Both rows and columns undergo clustering, grouping genes and samples with similar expression patterns together. Each heatmap is titled with the comparison group, while the annotation colors for rows and columns represent the sample phenotypes. **D**, **E** The graphics display the enriched pathways in subgroups A and B, respectively. Each graph includes the enrichment curve and key statistical information for the specified pathways. The enrichment curve illustrates the enrichment status of genes within the gene set, with the x-axis representing the ranking of genes within the gene set, and the y-axis representing the enrichment score. Significant peaks in the enrichment curve indicate significant enrichment of pathways in the specified subgroup

The clinical variables in groups A and B were visualized (Fig. 3A), and it was found that groups A and B had significant differences.

### Different characteristics of biological behavior between two subgroups

**Fig. 4:**
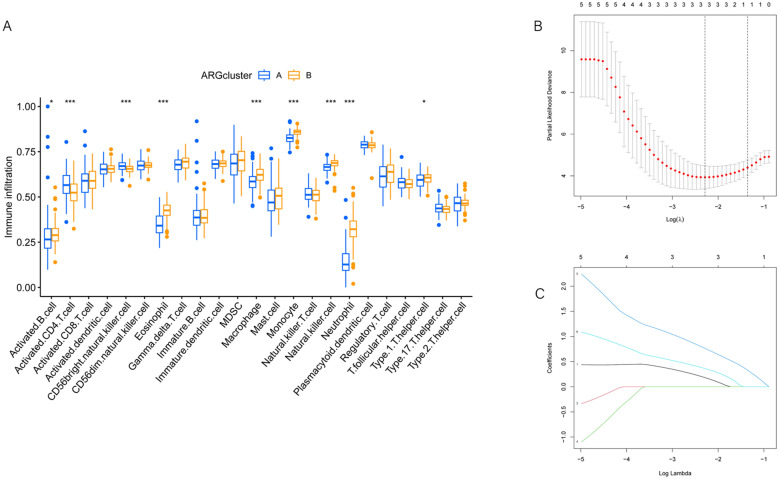
**A** The boxplot visualizes the distribution of immune infiltration scores for different types of immune cells, contrasting the immune infiltration between subgroups A and B. It also displays the significant level of immune infiltration differences between the two subgroups. **B** The cross-validation curve displays the cross-validation error at different lambda values. The x-axis represents the lambda values, while the y-axis represents the cross-validation error. **C** The Lambda curve illustrates the relationship between the regularization parameter (lambda) of the model and the sparsity of the model. The x-axis represents the value of lambda, where a higher lambda implies stronger regularization and coefficients tend toward zero. The y-axis represents the size of the model coefficients, which gradually approach zero as lambda increases. The points on the curve indicate the size of model coefficients for each lambda value, aiding in the selection of an appropriate regularization parameter

We conducted gene set variation analysis (GSVA) to explore distinct biological processes between the two subgroups. The analysis aimed to identify differential KEGG pathways and GOBP pathways in both subgroups. The results revealed significant enrichment of KEGG pathways in subgroup B (Fig. 3B), along with enrichment in GOBP pathways (Fig. 3C). Further examination of KEGG pathway enrichment in each subgroup separately (Fig. 3D, E) highlighted the RETINOL_METABOLISM pathway enrichment in subgroup B and the OLFACTORY_TRANSDUCTION pathway enrichment in subgroup A. Additionally, single-sample gene set enrichment analysis (ssGSEA) was employed to explore immune infiltration levels between the two subgroups. The analysis confirmed significantly higher immune cell abundance in subgroup A compared to subgroup B, demonstrating distinct immune infiltration patterns (Fig. 4A). These findings underscore differential characteristics between the two subgroups in terms of KEGG pathways, GOBP pathways, and immune infiltration levels.

### Construction of anoikis-related prognostic risk model

**Fig. 5:**
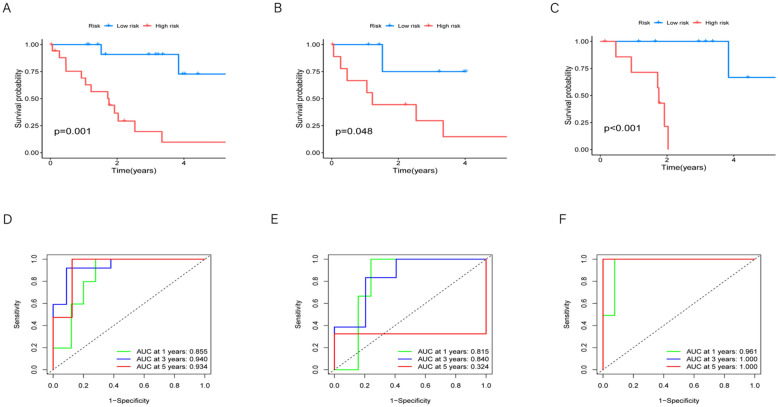
**A** Survival curves for all samples, with time (in years) on the horizontal axis and survival probability on the vertical axis. Curves are represented in different colors indicating survival curves for high and low-risk groups, with survival differences' p-values annotated below the curves. **B** Survival curve plot for the validation group. **C** Survival curve plot for the training group. **D** ROC curves for all samples. Three ROC curves are plotted in the graph, representing time points at 1 year, 3 years, and 5 years. Each curve is colored green, blue, or red, respectively. The legend displays the AUC values for each time point. **E** Validation group ROC curve. **F** ROC curves for the training group

To construct a prognostic risk model related to cholangiocarcinoma, we randomly selected 16 samples from the ICC dataset for the training dataset and 16 samples for the testing dataset. Previously, we applied univariate Cox proportional hazard regression analysis to screen candidate prognostic genes using 125 genes and identified 6 genes based on *p* < 0.05 (Fig. 1C). To refine the variables, we applied LASSO regression to these genes and ultimately determined 3 genes (Fig. 4B, C). Subsequently, we divided patients into two groups based on the median risk score. We analyzed the three groups separately: total, training, and validation groups. In the validation group, patients with higher risk scores generally had lower survival probabilities and died earlier compared to those with lower risk scores (Fig. 5B). Additionally, patients with higher risk scores in both the total and training datasets also had lower survival probabilities and died earlier compared to those with lower risk scores (Fig. 5A, C).

**Fig. 6:**
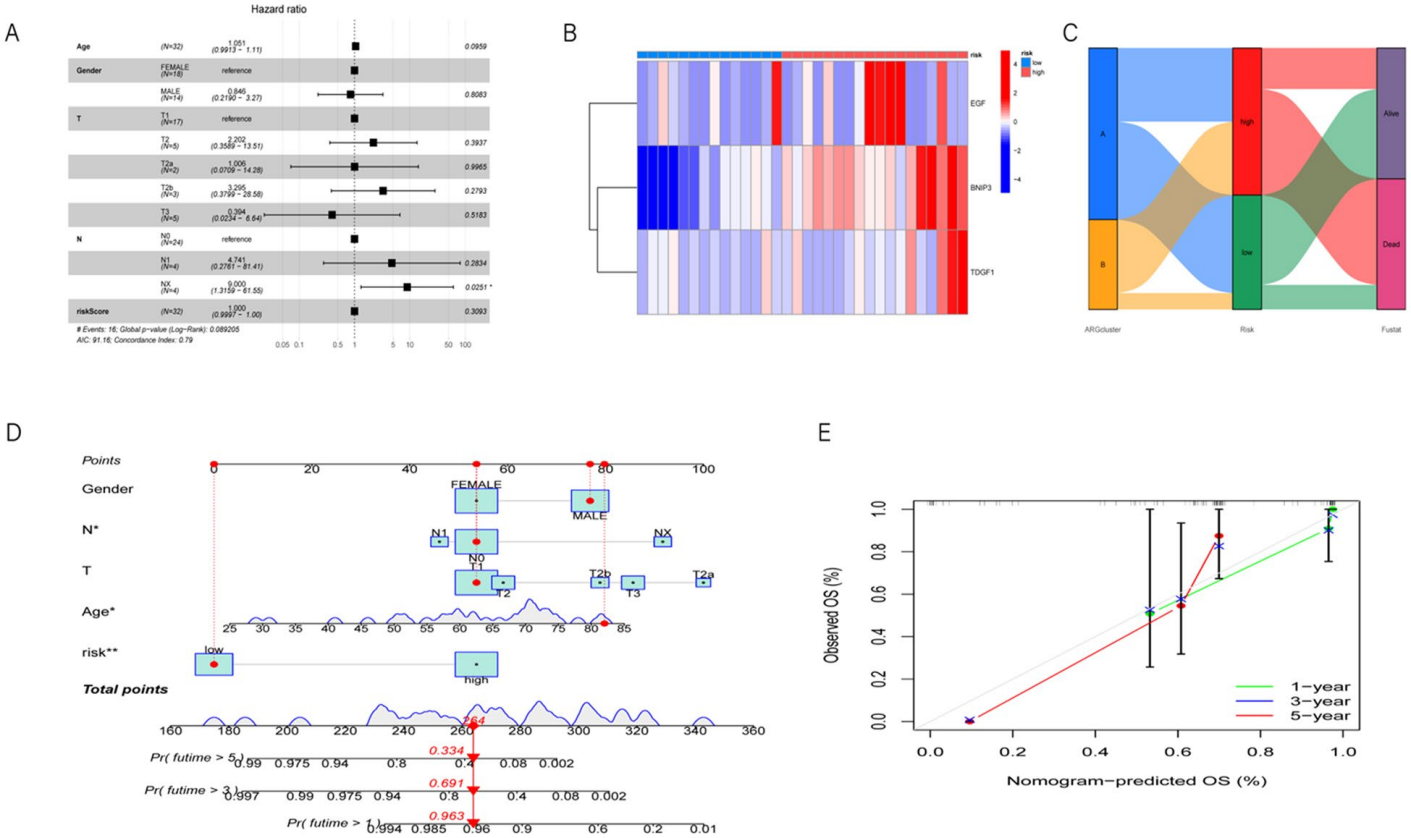
**A** The forest plot illustrates the hazard ratios of various variables, where each small square represents a variable. The horizontal position corresponds to the hazard ratio of the variable, while the line represents the 95% confidence interval. Longer lines indicate larger confidence intervals, reflecting greater uncertainty. **B** The heatmap illustrates the relationship of risk scores among samples, where rows represent samples. Samples are divided into two groups based on risk values: low risk is indicated by blue color, while high risk is depicted by red color. Columns represent genes. The intensity of color reflects the level of risk score, with darker colors indicating higher risk and lighter shades suggesting lower risk. **C** The boxplot shows the association between genomic clustering, risk score, and survival status. **D** Nomograph predicts survival. **E** Nomograph calibration curve

Furthermore, we predicted the overall survival rates in the three datasets. At 1 year, 3 years, and 5 years, the area under the time-dependent ROC curve (AUC) in the total dataset was 0.855, 0.940, and 0.934, respectively (Fig. 5D). In the validation dataset, the corresponding AUCs were 0.815, 0.840, and 0.324 (Fig. 5E), while in the training dataset, they were 0.961, 1.000, and 1.000 (Fig. 5F). In both the training and testing datasets, the expression of prognostic genes was significantly upregulated in high-risk patients compared to low-risk patients (Fig. 6A).

### Validation of anoikis-related prognostic risk model

We merged the risk data with clinical factors for subsequent analysis. The merged dataset included information on survival time, event status, clinical factors, and risk scores. We performed independent prognostic analysis using Cox proportional hazard models on the merged data and generated a forest plot (Fig. 6A). The study results indicated that the risk score could serve as a reliable and independent indicator for assessing patient prognosis. Subsequently, we further combined the data from the three genes (EGF, BNIP3, TDGF1) obtained from multivariable Cox proportional hazard regression analysis and our established high- and low-risk groups. We conducted gene expression analysis and obtained a risk heatmap (Fig. 6B), revealing high expression of EGF, BNIP3, and TDGF1 in the high-risk group, while low expression was observed in the low-risk group. Finally, we compared the mean risk scores between subtypes A and B. Using t-tests to examine the differences in means between the A and B subgroups and between the high- and low-risk groups, we identified the distribution and relationship between the two subgroups and two risk groups (Fig. 6C). The results confirmed that our predictive model grouping could effectively predict patient risk and survival rates.

### Constructing a risk nomogram

**Fig. 7:**
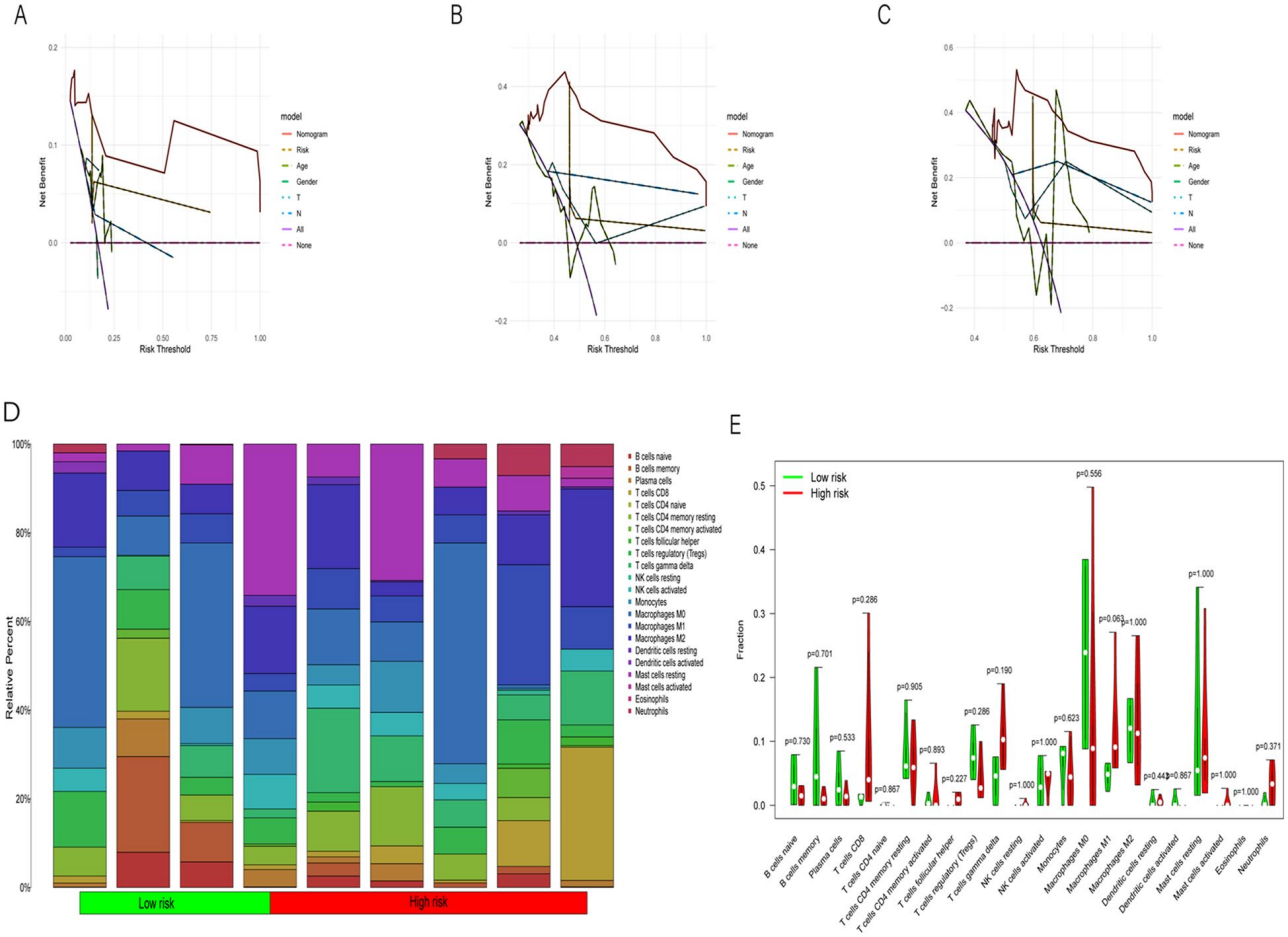
**A** This graph displays the results of decision curve analysis at the 1-year time point. The x-axis represents the threshold, which is the probability threshold used for predicting event occurrences. The y-axis represents the net benefit, calculated as the weighted sum of true positive rates minus the sum of false positive and false negative rates. Each curve represents a predictive model, distinguished by different line types according to the model type. **B** The decision curve analysis results at the 3-year time point are depicted in this graph. **C** The decision curve analysis results at the 5-year time point are depicted in this graph. **D** The bar plot depicts the relative percentages of each immune cell infiltration type. Different colors distinguish between the low-risk and high-risk groups. This visualization enables a straightforward comparison of the proportions of immune cell infiltrations between the low-risk and high-risk groups. **E** The violin plot illustrates the distribution of each immune cell infiltration type in both the low-risk and high-risk groups. It provides a visual comparison of the distribution differences between the two groups across various immune cell infiltration types. Wilcoxon rank-sum test was utilized to determine the statistical significance between the low-risk and high-risk groups, and the significance levels are annotated on the plot

Based on the risk scores and clinical variables constructed earlier, we developed a calibration plot to quantitatively predict patients' survival probabilities at 1, 3, and 5 years (Fig. 6D). Using the calibration plot, we can forecast patients' survival rates. For example, a 54-year-old female patient diagnosed clinically with ICC, T1N0, and high risk has a total score of 264 according to the model. Her survival probabilities at 1, 3, and 5 years are 96.3%, 69.1%, and 33.4%, respectively. It is worth noting that the prognosis of ICC is generally poor. The calibration curve demonstrates its good reliability (Fig. 6E). The decision curve analysis (DCA) at 1, 3, and 5 years (Figs. 7A–C) indicates that the risk score serves as a valuable predictor of survival rates for ICC patients.

### Immune infiltration in different risk groups

**Fig. 8:**
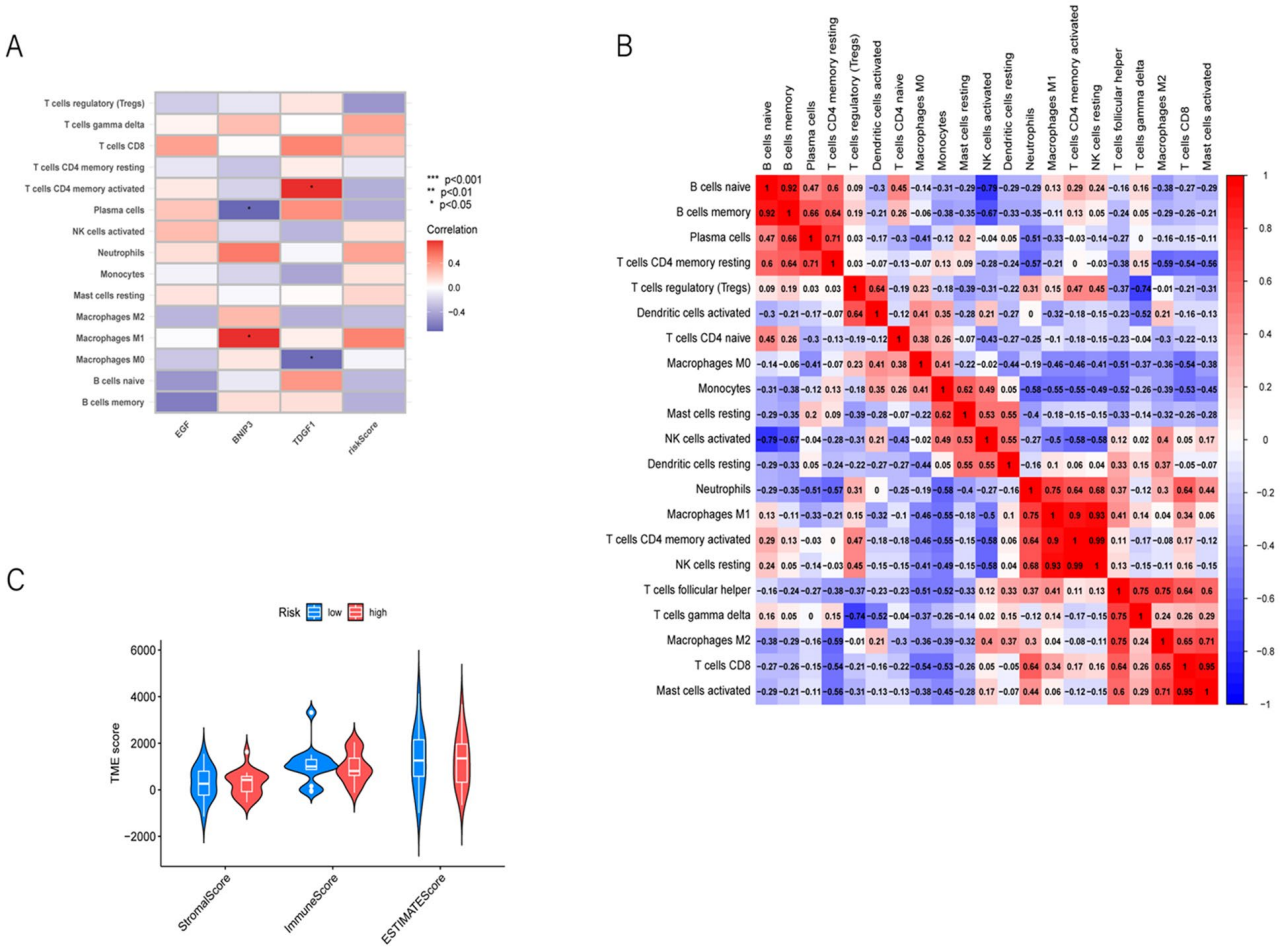
**A** The correlation heatmap illustrates the correlation between genes and immune cell infiltration types. Darker colors indicate stronger correlations, with red indicating positive correlations and blue indicating negative correlations. Significance levels of correlations are annotated in the graph. **B** The correlation heatmap displays the correlations between different types of immune cell infiltrations. It visually represents the strength and direction of correlations through color variations, where positive correlations are depicted in red and negative correlations are depicted in blue. **C** The violin plot illustrates the distribution of low-risk and high-risk groups across different types of tumor microenvironment scores

The development of intrahepatic cholangiocarcinoma (ICC) and the effectiveness of immunotherapy are significantly influenced by the tumor immune microenvironment (TME). To address this, we examined the TME of ICC patients more closely. We used "CIBERSORT" to measure the relative proportions of infiltrating immune cells in the high-risk and low-risk groups. Firstly, we sorted the samples' risk scores from low to high to display the proportions of various immune cells (Fig. 7D). The infiltration of monocytes and mast cells was greater in the low-risk group (Fig. 7E), suggesting a potential role of mast cells in inhibiting poor prognosis in ICC. The three genes used to construct the risk score were closely associated with many immune cells (Fig. 8A). We also conducted a correlation analysis between immune cell infiltration and risk scores, generating a heatmap of the correlation. (Fig. 8B). Furthermore, we visualized the tumor microenvironment score and compared and grouped the scores based on risk levels to further analyze the relationship between the tumor microenvironment and risk (Fig. 8C).

### Correlation analysis of ANRGs and tumor immune microenvironment

**Fig. 9:**
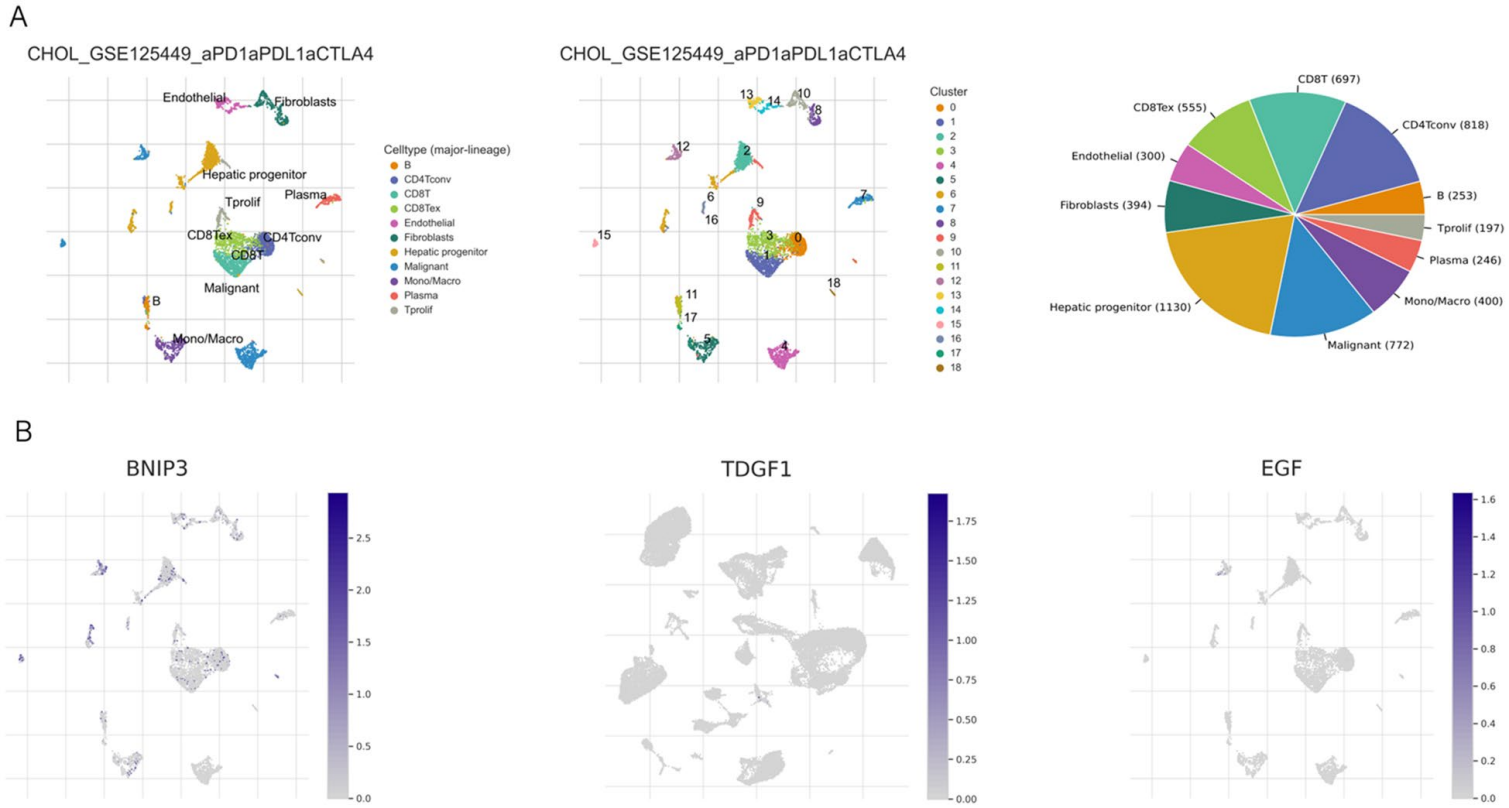
**A** The annotation of all cell types and the percentage of each cell type in GSE125449. **B** The expression of EGF, BNIP3, and TDGF1 in each cell type

To detect the expression of three ANRGs in the tumor microenvironment (TME), we utilized the single-cell dataset GSE125449 from the TISCH database for CHOL. The GSE70630 dataset comprises 10 cell clusters and 4 intermediate cell types, as illustrated in Fig. 9A, depicting their distribution and abundance. From the Fig. 9B, it can be observed that BINP3 is expressed in multiple cell clusters, such as CD4+ T cells, CD8+ T cells, Hepatic progenitors, and Mono/macrophages.

## Discussion

In this study, we identified the role of anoikis-related genes in the development and progression of intrahepatic cholangiocarcinoma and constructed a prognostic risk model. We also explored the correlation between infiltrating immune cells and prognostic genes and risk scores. TCGA samples were divided into two subgroups, with subgroup A having a higher survival probability. To explore the differences in biological processes between the two subgroups, we conducted Gene Set Variation Analysis (GSVA) to identify different KEGG pathways and COBP pathways in the two subgroups. The results indicated a high enrichment of the KEGG pathways in subgroup B. Subgroup B also showed enrichment in COBP pathways. We further analyzed the enrichment of KEGG pathways in the two subgroups separately and found that the RETINOL_METABOLISM pathway was enriched in subgroup B, while the OLFACTORY_TRANSDUCTION pathway was enriched in subgroup A. Additionally, we applied the single-sample gene set enrichment analysis (ssGSEA) algorithm to explore the level of immune infiltration between the two subgroups. We confirmed that compared to subgroup B, subgroup A had significantly higher immune cell abundance, exhibiting a distinct immune infiltration pattern, including eosinophils, macrophages, monocytes, natural killer cells, neutrophils, and type 1 helper T cells. Through weighted gene co-expression network analysis (WGCNA), we screened out genes highly associated with hepatocellular carcinoma and constructed a prognostic risk model. Finally, we analyzed the correlation between infiltrating immune cells and prognostic genes and risk scores. We found that the risk score was significantly positively correlated with most immune cells, and prognostic genes were highly correlated with most immune cells. In conclusion, our findings suggest that anoikis-related genes can be used to assess the prognostic significance of intrahepatic cholangiocarcinoma and the potential for immune therapy.

According to current research progress, we found that some signaling pathways play an important role in the biology of ICCA and may be related to the activation of anoikis-related genes. For example, the Ras-MAPK signaling pathway is one of the most important pathways in ICCA and has been widely reported in some studies. Activation of this pathway is related to the promotion of cell proliferation and survival and may be related to anoikis resistance [23]. In addition, the Hippo signaling pathway also plays a role in ICCA, which plays a key role in regulating hepatocyte proliferation and apoptosis [24]. Understanding the mechanism of this pathway is crucial for the development of new targeted therapies. The results from GSVA indicate that the RETINOL_METABOLISM pathway is enriched in subgroup B. Previous studies have already confirmed the presence of abnormal retinol metabolism in the progression of prostate cancer [25], single nucleotide polymorphisms (SNPs) in genes related to the retinol metabolism pathway are also associated with the occurrence of breast cancer and pancreatic cancer [26, 27]. The OLFACTORY_TRANSDUCTION pathway is enriched in subgroup A, previous studies found that the 301 olfactory receptor genes showed different expression patterns in 968 cancer cell lines derived from different cancer types, olfactory receptors have the ability to sense organic molecules and mediate signaling for survival and migration stimuli, enabling them to function in cancer cells [28–30]. The abnormal activation of certain signaling pathways is a key factor in the development of cancer [31]. This is consistent with the increased risk of death observed in subgroup B, where the abnormal activation of these pathways is more pronounced compared to Group A. These results indicate that an in-depth study of the abnormal activation of anoikis-related signaling pathways in ICCA is of great significance for understanding the disease mechanism of ICCA and developing new treatment strategies. Further studies are needed to further elucidate how these pathways influence ICCA development and whether they may serve as novel therapeutic targets.

Recently, numerous studies have shown that tumor-associated immune cells play an important role in regulating cancer. Immune cells are the cellular basis of cancer immunotherapy [32]. In our study, the abundance of immune cells in subpopulation A is significantly higher than that in subpopulation B and has a unique immune infiltration pattern, including eosinophils, macrophages, monocytes, natural killer cells, neutrophils, neutrophils, Type 1 neutrophils, and CD4 T cells. In addition, the prognosis of subgroup A is better than that of subgroup B, which indicates that there is some imbalance in the immune system of subgroup B, causing an immune desert [33]and allowing cancer cells to grow wildly. Research results have confirmed that activated eosinophils have bactericidal and T-cell regulatory activities and express costimulatory molecules CD80 and PD-L1 [34]. In addition to this, tumor microenvironment-infiltrated monocyte-derived cells have been shown to induce antitumor effects [35, 36], some experiments have shown that NK cells can distinguish abnormal cells from healthy cells, produce more specific anti-cancer toxicity, and reduce unnecessary complications [37, 38]. Also, the function of NK cells in adoptive tumor immunotherapy cannot be ignored. Studies have confirmed that CAR T cell-related cytokine release syndrome (CRS) and neurotoxicity are an important issue in clinical practice, especially those involving Side effects of T cells, whereas memory NK cells pre-complexed with agonist molecules provide an alternative approach to achieve CAR-like specificity without the need for lengthy manufacturing and reduce the occurrence of CRS to a greater extent [39].In mice with mouse breast cancer virus promoter-driven polyomavirus intermediate T antigen (MMTVPyMT) or MMTV-myc breast cancer xenograft tumors, neutrophils may exert a cytotoxic effect by producing H2O2, thereby inhibiting tumor growth [40, 41]. Moreover, previous studies have demonstrated that CD4+ T cells and eosinophils cooperate to enhance the response of breast cancer to immune checkpoint blockade [42], and that in bladder cancer, cytotoxic CD4 T cells can kill autologous tumors in an MHC class II-dependent manner [43]. However, in the high-risk group, due to significant immunosuppression, oncolytic virus combined with immunotherapy may be considered. It has been demonstrated that pretreatment of established tumors with oncolytic viruses may shape the local tumor microenvironment to improve T cell recruitment and effector function [44]. The results of the immune infiltration analysis also indicated that the low-risk group had a positive prognostic capability compared with the high-risk group. Consequently, our categorization effectively highlights the potential for deriving benefits from immunotherapy.

In intrahepatic cholangiocarcinoma (ICCA), abnormal activation of anoikis-related genes may be a key factor leading to disease progression. In our study, we found that three novel prognostic genes (EGF, BNIP3, TDGF1) were risk factors for anoikis resistance in ICCA. Epidermal growth factor (EGF) is a growth factor that plays an important role in cell proliferation, survival and migration by binding to its receptor EGFR [45]. EGF signaling enhances anoikis resistance through both direct survival pathways and regulation of cell–matrix adhesion molecules, which is crucial for cancer cell survival during metastasis. EGF is highly expressed in liver cancer tissues and is positively correlated with tumor grade [46]. A group of studies found that overexpression of EGF leads to prolonged survival of liver cancer cells and resistance to anoikis [47]. Mechanistically, EGF activates multiple downstream signaling pathways including Ras/Raf/MEK/ERK and PI3K/Akt pathways through binding to EGFR. These pathways promote the expression of anti-apoptotic proteins like Bcl-2 while suppressing pro-apoptotic proteins, thereby enhancing anoikis resistance [48]. Additionally, EGF signaling regulates cell adhesion and migration by modulating cell surface adhesion molecules, which enables cancer cells to survive after detachment from the extracellular matrix [49]. Bcl-2/adenovirus E1B 19 kDa interacting protein 3 (BNIP3) is a mitochondrial member of the Bcl-2 protein family containing only the BH3 domain. BNIP3, as a mitochondrial protein, regulates cell survival primarily through modulating mitochondrial function and membrane potential, affecting the release of cytochrome c and subsequent apoptotic signaling [50]. BNIP3 is activated by hypoxia and mainly functions as a cell death regulator under hypoxic conditions [51]. In liver cancer cells, BNIP3 is considered a therapeutic target for cancer metastasis because upregulation of BNIP3 increases the anti-tumor ability of liver cancer cells [52]. Our single-cell analysis revealed that BNIP3 is expressed in multiple immune cell populations, including CD4+ T cells, CD8+ T cells, and macrophages, suggesting its potential role in modulating the tumor immune microenvironment. Moreover, cancer cells with upregulated BNIP3 may possess an immune evasion arsenal. Treatment with epidermal growth factor (EGF) and insulin-like growth factor (IGF) suppressed BNIP3-induced cell death in hypoxic conditions. Conversely, downregulating EGF receptor signaling with Erb2 antibody increased cell death under hypoxic conditions [53]. The human teratoma-derived growth factor 1 gene (TDGF1) belongs to the recessive FRL1 gene family encoding epidermal growth factor and was originally isolated from human teratoma [54]. Previous in vitro and in vivo studies have shown that TDGF1 functions as an oncogene, regulating signaling pathways and cellular mechanisms [55–58]. In the context of breast tumors, TDGF1 has been observed to be associated with the molecular mechanism that leads to the loss of adherens junctions, namely epithelial-mesenchymal transition, which plays an important role in and can lead to tumor invasion and metastasis [58–60]. This process plays a significant role in tumor invasion and metastasis. This mechanism has been demonstrated in other cancer species, suggesting that TDGF1 may promote anoikis resistance in papillary thyroid cancer by enabling cancer cells to survive independently of anchoring during metastasis [61]. However, it is important to emphasize that while ICCA may have a similar mechanism, further experiments are needed to verify its role in ICCA invasion and metastasis. Collectively, these three genes appear to form an intricate network regulating anoikis resistance and immune responses in ICCA. While EGF primarily mediates survival signaling and cell adhesion, BNIP3 functions as a key regulator of cell death and immune cell interactions, and TDGF1 promotes metastatic potential through EMT regulation. A more thorough understanding of these mechanisms has the potential to provide new therapeutic targets for ICCA treatment, though further experimental validation is needed to fully elucidate their interactions and clinical applications.

In summary, our comprehensive analysis provides novel insights into the role of anoikis-related genes in ICCA progression and their relationship with the tumor immune microenvironment. The identification of three key genes (EGF, BNIP3, TDGF1) provides novel therapeutic targets and prognostic indicators. In particular, EGF's involvement in the EGFR signaling pathway suggests potential synergy with existing targeted therapies, while BNIP3's role in cell death regulation may influence treatment response. Integrating multiple signaling pathways, including Wnt/β-catenin, EGF/EGFR, and TGF-β1, demonstrates the complex molecular network that governs anoikis resistance. These pathways interact with different immune cell populations, in particular tumor-associated macrophages and regulatory T cells, creating a sophisticated immune microenvironment that influences disease progression and treatment outcome. Our findings suggest that targeting anoikis resistance mechanisms may provide novel therapeutic opportunities. The correlation between the risk score and immune cell infiltration patterns suggests potential applications in immunotherapy response prediction and patient stratification.

Several limitations of our study should be considered. First, the relatively small sample size may limit the statistical power and generalisability of our findings. Multi-center validation studies with larger cohorts are needed to confirm the prognostic value of our gene signature. Second, the lack of comprehensive experimental validation, especially in vitro and in vivo functional studies, limits our understanding of the precise molecular mechanisms. Future research should include CRISPR-based functional studies and patient-derived xenograft models to validate the role of the identified genes in anoikis resistance and tumor progression. Third, although our prognostic model is promising, its clinical utility requires validation in prospective studies with diverse patient populations. Integrating additional clinicopathological features and treatment response data may improve the predictive accuracy of the model. Despite these limitations, our findings provide a strong foundation for future research directions in ICCA treatment. Integrating multi-omics approaches will likely provide deeper insights into the molecular mechanisms of anoikis resistance. Novel therapeutic strategies combining pathway inhibitors with immunotherapy may offer enhanced therapeutic efficacy.

Future studies should focus on validating our findings in larger cohorts and investigating the functional relationships between identified genes and immune cell populations. The development of biomarker-driven clinical trials targeting anoikis resistance mechanisms may lead to more effective personalized treatment approaches. The potential role of these anoikis-related genes (EGF, BNIP3, TDGF1) as therapeutic targets warrants further investigation, especially in combination with existing treatments. Understanding the complex interplay between anoikis resistance and the immune microenvironment may reveal new opportunities for therapeutic intervention in ICCA.

## Conclusions

The research findings indicate that we have successfully classified patients with ICCA into two subgroups, and identified subgroup B with lower survival probability. We identified three prognostic genes related to anoikis (EGF, BNIP3, TDGF1), which have been shown to be closely related to anoikis resistance. The prognostic risk model effectively predicts patients' overall survival rates and has been validated in ICGC and GEO datasets. Additionally, there is a significant correlation between infiltrating immune cells and prognostic genes and risk scores, indicating the crucial role of immune regulation in the development of ICCA. In conclusion, our study identified ICCA subgroups and prognostic genes related to anoikis, highlighted the clinical significance of anoikis-related genes, and may contribute to the development of immunotherapy strategies for ICCA patients. However, further clinical trials are needed to validate these findings.

## Data Availability

The data source for this study is provided in the manuscript.
